# A Digital Mask-Voiceprint System for Postpandemic Surveillance and Tracing Based on the STRONG Strategy

**DOI:** 10.2196/44795

**Published:** 2023-11-06

**Authors:** Xiaogao Pan, Alphonse Houssou Hounye, Yuqi Zhao, Cong Cao, Jiaoju Wang, Mimi Venunye Abidi, Muzhou Hou, Li Xiong, Xiangping Chai

**Affiliations:** 1 Department of Emergency Medicine Second Xiangya Hospital Central South University Changsha China; 2 Emergency Medicine and Difficult Diseases Institute Central South University Changsha China; 3 School of Mathematics and Statistics Central South University Changsha China; 4 Department of Gastroenterology Second Xiangya Hospital Central South University Changsha China; 5 General Surgery Department Second Xiangya Hospital Central South University Changsha China

**Keywords:** COVID-19, surveillance, digital tracing, mask management, voiceprint, Spatiotemporal Reporting Over Network and GPS, STRONG, STRONG strategy, living with the virus, dynamic clearance, surveillance, digital surveillance, pandemic, vaccine, public health, mental, social, communication technology, communication, tracing

## Abstract

Lockdowns and border closures due to COVID-19 imposed mental, social, and financial hardships in many societies. Living with the virus and resuming normal life are increasingly being advocated due to decreasing virus severity and widespread vaccine coverage. However, current trends indicate a continued absence of effective contingency plans to stop the next more virulent variant of the pandemic. The COVID-19–related mask waste crisis has also caused serious environmental problems and virus spreads. It is timely and important to consider how to precisely implement surveillance for the dynamic clearance of COVID-19 and how to efficiently manage discarded masks to minimize disease transmission and environmental hazards. In this viewpoint, we sought to address this issue by proposing an appropriate strategy for intelligent surveillance of infected cases and centralized management of mask waste. Such an intelligent strategy against COVID-19, consisting of wearable mask sample collectors (masklect) and voiceprints and based on the STRONG (Spatiotemporal Reporting Over Network and GPS) strategy, could enable the resumption of social activities and economic recovery and ensure a safe public health environment sustainably.

## Introduction

SARS-CoV-2, the virus responsible for COVID-19, has caused a global pandemic, making it the most significant and devastating disaster of the century [1]. Lockdowns and border closures imposed mental, social, and financial hardships in many societies. Even investing trillions of dollars, multiple virus attacks are difficult to stop, as vaccines have always been slower than mutations, and regional imbalances in medical care have also severely hampered vaccine coverage [2]. Despite the reduced severity of Omicron compared to the Wuhan strain, people are still not fully prepared to live with the virus, especially older people, immunocompromised persons, and persons with comorbidities. Struggling with the difficult choice between reopening and lockdown, there remains an absence of global consensus on cost-effective testing strategies and sound public health measures that would enable the control of community infections and minimize disruptions to society and the economy [3].

In current perceptions, a mask is viewed as a simple tool for effectively interrupting the transmission chain by blocking the spread of SARS-CoV-2 droplets and filtering the aerosols produced by the virus [4]. However, a large number of discarded masks aggravated environmental problems and caused secondary transmission or cross-infection. Despite continuous attempts to improve masks, which are well described in previous reports [5], it is not enough to simply improve traditional masks’ filtration, protection, and biodegradability. Only updating functions without innovative concepts may not be the real mask revolution in the digital age. Notwithstanding the strong resistance to masks by some pursuers of bodily autonomy [6], we still believe that masks are effective as a public health tool, but some improvements should be emphasized to better align with the current situation of global epidemic prevention.

Current trends still need pinpoint surveillance to achieve dynamic clearances, with the only lacking component being an effective program [1]. Imagine a scenario where the wearer’s infection status can be quickly detected, information about the infected individual can be collected synchronously, and digital contact tracing can locate places accurately at the same time. All these possibilities could be achievable by using masks, thus potentially sparking a digital mask revolution. If superspreading individuals or events can be systematically identified, control efforts may reasonably focus on mitigating transmission in a more targeted manner [7]. This necessitates a high-performance digital surveillance system complementing mobile and wearable mask sample collectors, referred to as the masklect (for this purpose, we collected and tested SARS-CoV-2 RNA on surgical masks worn by COVID-19–infected patients from China; part 1 in Multimedia Appendix 1) [4,5,8-20]. According to our previous reports [8], it is feasible to use GPS and geospatial artificial intelligence (AI) technology from smartphones to collect personal spatiotemporal trajectory data to construct the epidemic prevention strategy—STRONG (Spatiotemporal Reporting Over Network and GPS) [8]. This strategy has been previously used in systems like Google Flu Trends and the South Korean contact tracing system for COVID-19 [21,22]. Modeling studies suggest that contact tracing had the potential to slow the spread of the virus in the presence of relaxed lockdown measures [23]. Nevertheless, traditional digital contact tracing is only used to build an epidemic prevention network, as it cannot directly identify the user’s infection status. In some cases, a health QR code may not necessarily represent a “healthy sign,” as observed in China. With the wide spread of COVID-19, more scholars are calling for a digital surveillance system against future pandemics [7,22,24,25]. Therefore, being equipped with rapid detection tools, such as masklect, would be helpful in reducing recall bias and infection identification interval. Notably, although masklect detection is partly designed to enhance the detection of asymptomatic or presymptomatic cases, it is interesting to monitor changes in voiceprints before and after infection. This approach can help in the early detection of COVID-19 in individuals, especially those experiencing subtle symptoms, such as dry cough, hoarseness, or pharyngeal discomfort. Unlike the Massachusetts Institute of Technology’s AI technology for identifying coughs [26], the AI-based voiceprint health code installed in smartphone apps will be matched to personal spatiotemporal track data. With intelligent computing and integrated analysis, it is easy to assess changes in users’ voiceprints and the voiceprint of those who have had temporal and spatial intersections with the user [27]. If multiple people in the time-space intersection have voiceprint anomalies one after another, it can be inferred that they are caused by the spread of the virus.

As the severity of the virus diminishes, countries are transitioning from a pandemic response mode to living with the virus, whereby the main role of testing will shift from diagnosis and case detection to surveillance [9,28,29]. Unfortunately, with the self-test tool promotion, the current system still relies on patients self-reporting to health care workers to control the spread of the virus [7,22]. More than 20%-40% of virus transmission can be attributed to individuals who are asymptomatic or presymptomatic before the nasopharyngeal swabs show positive test results [4,30]. The strategies to interrupt the transmission chains within communities by scaling up testing, contact tracing, and isolation are still subject to many restrictions [9]. The COVID-19–related mask waste crisis has also caused serious environmental problems and virus spreads [10]. It is timely and important to consider how to precisely implement surveillance for the dynamic clearance of COVID-19 and how to efficiently manage discarded masks to minimize disease transmission and environmental hazards. Based on these concerns, we sought to propose an appropriate testing strategy that could enable the resumption of social activities and economic recovery and ensure a safe and sustainable public health environment. Therefore, a digital surveillance system consisting of masklect and voiceprints to combat COVID-19, based on the STRONG strategy, may be feasible to more efficiently prevent and control SARS-CoV-2 and reduce environmental crisis and ecological issues caused by mask waste in the future.

## MV-STRONG Program for Intelligent Surveillance of Infected Cases and Centralized Management of Mask Waste

A novel digital surveillance system was introduced based on the STRONG strategy; this system was equipped with both masklect (M) and voiceprint (V) health codes, for the comprehensive prevention and control of COVID-19 (referred to as MV-STRONG). The MV-STRONG program consists of two streams in general: (1) population-based disease surveillance (ie, the use of lab testing for mask-based sample collection and diagnosis and the use of mobile phone apps to detect voiceprints of suspected or infected cases; (2) centralized mask-management to minimize the transmission of disease-related and environmental hazards.

### Part 1：Intelligent Tracking System Using Mask-Based Samples and Voiceprint Change Detection

**Figure 1:**
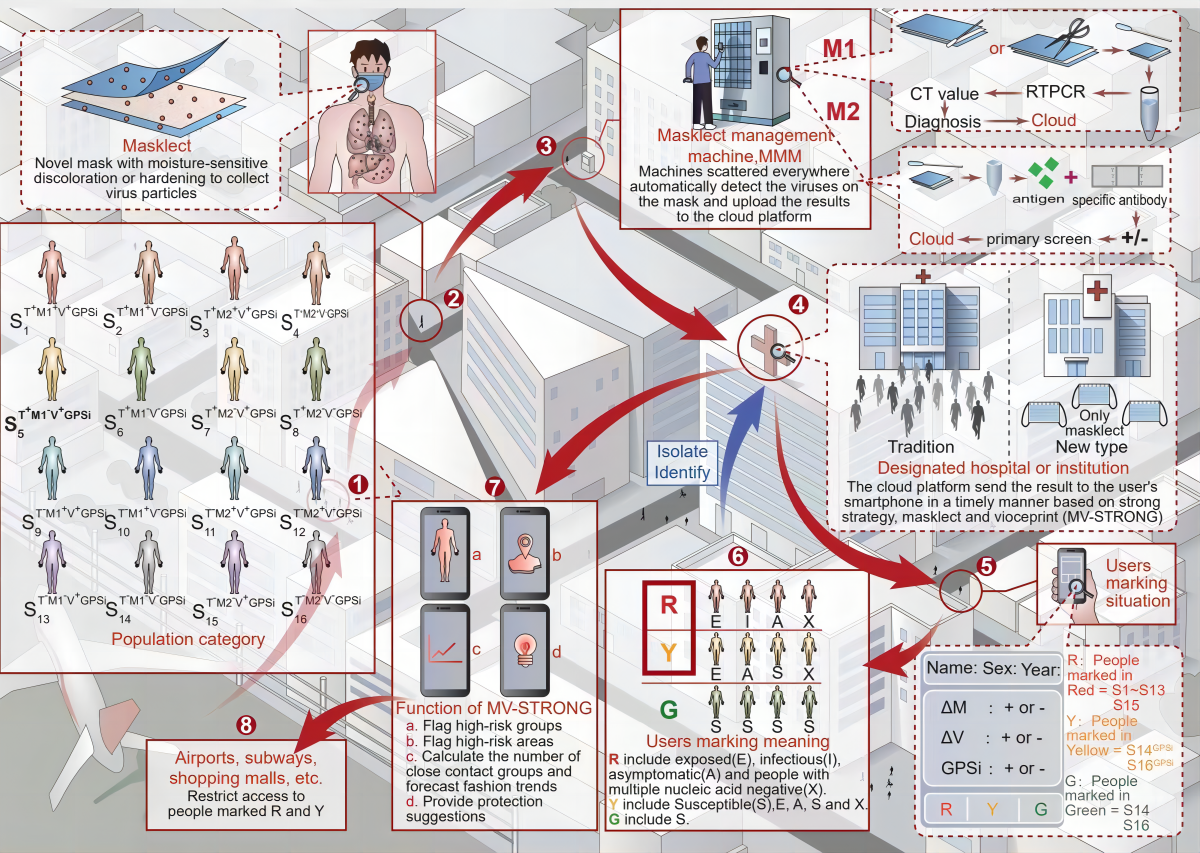
Panoramic representation of the MV-STRONG (mask and voiceprint-Spatiotemporal Reporting Over Network and GPS) system based on a future urban context. A: asymptomatic; CT: cycle threshold; E: exposed; G: green; GPSi: real-time GPS; I: infectious; M1: masklect nucleic acid test; M2: masklect antigen detection; MMM: mask management machine; R: red; RTPCR: reverse transcription polymerase chain reaction; S: susceptible; T: throat swabs nucleic acid test; V: voiceprints; X: people with multiple throat swabs with nucleic acid test negative; Y: yellow.

The operation of individuals and objects within the first part is based on a future urban context and consists of 8 steps that are fundamental to the running of the MV-STRONG program (Figure 1).

In step 1, people from all over the world are categorized into 16 different groups based on the negative or positive detection results, including throat swab nucleic acid tests, masklect nucleic acid tests, masklect antigen detection, voiceprints, and real-time GPS (GPSi). Throat swabs include nasopharyngeal swabs or oropharyngeal swabs, which are currently routine testing measures.

In step 2, masklect may represent a novel functional mask, which can enrich virus particles in the middle layer through the improvement of structure and material based on the filtration performance of the mask. Moisture-sensitive discoloration or hardening is even achieved to aid in distinguishing used masks (the design concept of the masklect can be found in part 2 in Multimedia Appendix 1). When the wearer performs daily activities, such as breathing, coughing, and talking, the masklect continuously collects virus samples from the upper and lower respiratory and alimentary canals.

In step 3, after sample collection, the masklect will be put into the mask management machine (MMM) for centralized collection, and at the same time, the wearer will scan the personal QR code of the MV-STRONG app to link their individual information and update test results. The masklect is then divided into 2 parts (M1 and M2), and the ATM-like MMM, which is scattered everywhere, automatically detects these samples (M1 for nucleic acid test and M2 for antigen detection). The design concept of MMM can be found in part 3 of Multimedia Appendix 1. At this point, the app can be used to detect changes in cough voiceprints (Δvoiceprint) at any time according to the wearer’s willingness. It is recommended to use repeated moderate coughs 3 times for the detection of voiceprint changes before and after recording and validation to facilitate voiceprint homogenization. The cough voiceprint combines with GPSi to form a voiceprint health code constructed into the voiceprint database. The design concept of the voiceprint health code can be found in part 4 in Multimedia Appendix 1.

In step 4, all these test results, including throat swabs nucleic acid test, masklect nucleic acid test, masklect antigen detection, voiceprints, and GPSi, will be uploaded to the cloud platform of the designated hospital or institution for intelligent review and publishing. Testing mask-based samples and monitoring changes in cough voiceprints will easily identify infected and potentially infected individuals based on the spatiotemporal interactions with people who have been diagnosed. The cloud platform then sends the result to the wearer’s smartphone app promptly.

In step 5, the wearers are marked in different colors, such as red, yellow, and green, according to the detection results. According to an epidemiological classification, red includes exposed, infectious, asymptomatic, and people with multiple throat swabs with a negative nucleic acid test. Yellow includes susceptible, exposed, asymptomatic, and people with multiple throat swabs with a negative nucleic acid test. Green includes susceptible individuals. Combined with testing information tied to individual QR codes and geographic information systems, these color tags will help to quickly track suspected or infected cases and minimize the risk of virus spread.

In step 6, red and yellow tags will be retested nearby to identify the final diagnosis and decide on hospitalization or at-home care, depending on the severity of the disease.

In step 7, the MV-STRONG system will provide various functions, including risk warning, protection suggestions, travel optimization, MMM navigation positioning, and isolation point display.

In step 8, airports, subways, and large shopping malls might restrict access of people marked with red and yellow tags until the risk is eliminated.

After these 8 steps, step 1 will be repeated along with the whole process. With this system in operation, more and more people marked by red and yellow tags would be selected for surveillance, while those with green tags would be safely left to keep the cycling process going (mathematical simulations for running the MV-STRONG system can be found in part 5 in Multimedia Appendix 1).

### Part 2：Centralized Management System for Mask Waste Based on Sample Collectors

**Figure 2:**
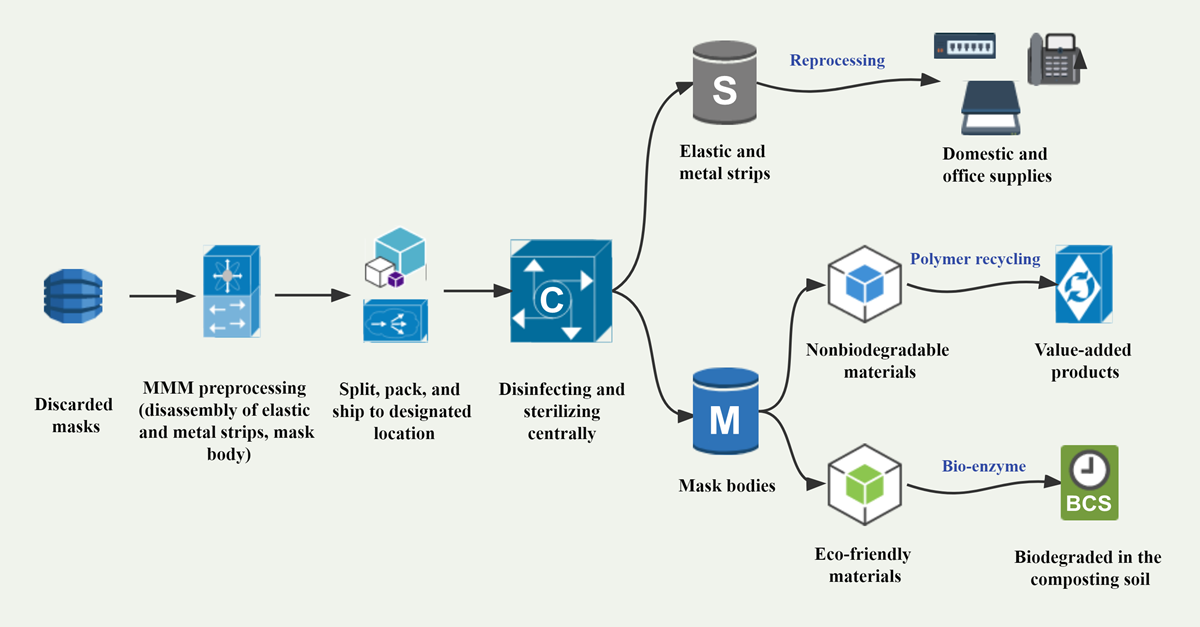
Centralized management system for mask waste based on sample collectors. BCS: biodegraded in the composting soil; C: disinfecting and sterilizing centrally; M: mask bodies; MMM: mask management machine; S: elastic and metal strips.

COVID-19 has driven a huge demand for masks and exacerbated the environmental issues associated with large volumes of mask waste [10]. Proper management of discarded masks, such as biodegradation and recycling, is timely and significant for achieving environmental sustainability. Part 2 of the MV-STRONG program, the eco-friendly disposal of discarded masks, may be an effective way to reduce the spread of disease and environmental hazards (Figure 2).

With the operation of the MV-STRONG program, a large number of masks are collected due to the MMM’s testing role, which creates a perfect way to manage mask waste centrally. The discarded masks are preprocessed after the MMM testing, mainly by disassembling the elastic and metal strips and the mask body, which are separated, packaged, and delivered to a designated location for centralized disinfection and sterilization. The disassembled elastic and metal strips are reprocessed into household and office supplies, such as nylon ropes and folders, while mask bodies, depending on the nature of the material, are disassembled into nondegradable or eco-friendly materials for polymer recycling into value-added products or for full degradation in composted soil. These treatments (reprocessing, polymer-recycling, and biodegradation) require high process costs based on current industrial technologies but are sustainable ways to address the waste crisis and ecological pollution in the long run.

## Discussion

With the wide spread of the Omicron variant, repeated pandemic attacks have exposed the defects and challenges in current epidemic containment efforts, namely the absence of adequate preparation during reopening phases and the lack of economic reserves during lockdown [29,31]. Living with the virus and resuming normal life are increasingly being advocated with decreasing virus severity and widespread vaccine coverage. Unfortunately, the current coexistence status indicates that neither the public nor the officials have taken countermeasures and contingency plans to stop the next more virulent variant of the pandemic [32]. Although vaccine and antiviral efforts continue to advance, pinpoint postpandemic surveillance is essential for living with SARS-CoV-2 in this new era of digital health [33,34].

In this viewpoint, we provided a postpandemic smart surveillance program that has the potential to fit the future COVID-19 response model. A distinct advantage of this intelligent surveillance system is its use of daily masks as sample collectors, voiceprint health codes as additional analysis, and geographic information systems as tracking tools; this approach enables the real-time reflection of the wearer’s infection status and the viral trajectory, thereby improving the monitoring of virus activity levels and superspreading events. When combined with genomics technology, this convenient sampling would provide access to information on viral mutations to develop targeted vaccines more quickly [35,36]. Another advantage of this surveillance system is that the mask-based intelligent testing assigns a digital code (personal QR code) to information about the virus’s transmission. This innovation of a digital mask concept would cleverly bypass the limitations of traditional masks and the complexity of multifunctional masks; it would combine medical internet technology to create a mask-based digital health signal [5]. Moreover, the sustainable management of masks will help to minimize massive waste crises and secondary health pollution in the future, which will have a positive effect on the reduction of global environmental problems and virus transmission [37,38].

**Figure 3:**
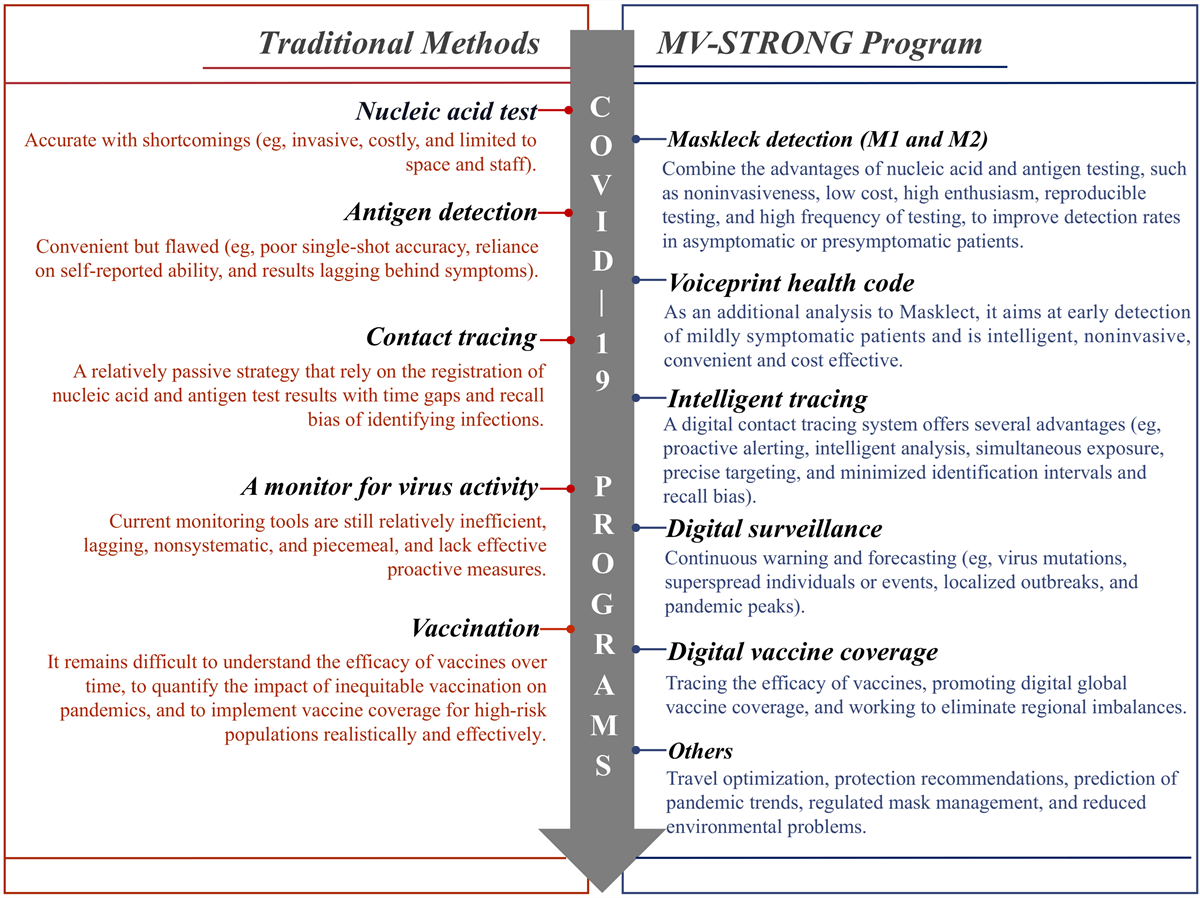
A detailed comparison between the traditional methods versus. MV-STRONG (mask and voiceprint-Spatiotemporal Reporting Over Network and GPS) program regarding COVID-19 control. M1: masklect nucleic acid test; M2: masklect antigen detection.

Similarly, a digital surveillance system would be more conducive to improving vaccine and medical efforts. If we could track patients’ vaccination dates, clinical evaluation, and underlying diseases, we could have near real-time insight into the efficacy of vaccines over time and better understand which viral and host characteristics contribute to breakthrough infections in immunized people. This intelligent system could combine patient vaccination information, clinical history, and COVID-19 status to implement digital vaccine coverage, thereby bridging the gap between public health and health care [39]. Such visual digital panels might also promote the equalization of medical resources and minimize discrimination and health care disparities among various ethnic groups and income levels [40,41]. In addition, MV-STRONG could model and predict the effective reproduction rate of SARS-CoV-2 via a contact index, enabling early warning. This surveillance system might minimize the risk of exposure and infection by translating the latest scientific knowledge and current public health policies into personalized recommendations [8]. Figure 3 provides a more detailed comparison between traditional methods and the MV-STRONG program in terms of COVID-19 control.

Nevertheless, there might be some inherent concerns, such as infringement of privacy, bodily autonomy, and the risk of abuse by a totalitarian party [42,43]. Indeed, it has been difficult to keep personal information private since the advent of the internet data era, especially in the context of COVID-19. The wide adoption of contact tracing and questionnaires (eg, Covapp) [44,45], web surveillance platforms, and health maps has also prompted the improvement of corresponding evaluation and control mechanisms, including privacy assessments, identification and traceability of infringement, and extended authorization. As it stands, these mechanisms have secured private data and provided a safe internet setting for tailoring public health policies to the local context [42]. We are fully confident that these potential pitfalls can be avoided through appropriate technology and legal constraints. Meanwhile, the public accessibility and coverage of the MMM and MV-STRONG systems need to be carefully considered and addressed, as lack of facilities in remote rural areas may lead to inefficiencies in the operation of the systems, which we hope will be well addressed in the future.

There are still several questions to facilitate the MV-STRONG development. Future science research is needed to explore a material to achieve viral enrichment and biodegradability of masks and to examine the sensitivity and specificity of detection by masks. 3D printing technology may be expected to mass-produce future masks [5], and developed industrial technologies are needed to reduce the cost of processing and recycling mask waste [46]. Information communication technology should be well prepared to evaluate the capabilities of digital contact tracing, ensuring that it is intelligent, capable of integrating multiple data sets and adaptable to various scenarios. Efforts should be made to ensure that personal privacy and data protection rights will not be breached or stolen and to create trustworthy, transparent, privacy-preserving digital contact-tracing technologies that are acceptable to populations [42]. Emphasizing mathematical simulations and epidemiological models is crucial for assessing the effect of the intelligent tracking system on COVID-19 and understanding its influence on economic recovery and political policies. To create sustainable diagnostic and surveillance systems, postpandemic investments should be increased to improve diagnostic testing capacity, coupled with information systems. Such systems will serve as the backbone of a health system, with data connectivity and appropriate technologies at every level.

## Appendix

Multimedia Appendix 1 Supplementary material, including the complete design concept of the mask, mask manager, and the vocal health code, as well as mathematical simulations and epidemiological models of the MV-STRONG (mask and voiceprint-Spatiotemporal Reporting Over Network and GPS) system under operational and nonoperational conditions.

## Abbreviations

AI: artificial intelligence
GPSi: real-time GPS
MMM: mask management machine
MV-STRONG: mask and voiceprint-Spatiotemporal Reporting Over Network and GPS
STRONG: Spatiotemporal Reporting Over Network and GPS
