# Supplementary material for: A Digital Mask-Voiceprint System for Postpandemic Surveillance and Tracing Based on the STRONG Strategy
Source: J Med Internet Res. 2023 Nov 6;25:e44795. doi: 10.2196/44795 (PMC10660213; doi:10.2196/44795)
Supplement: Multimedia Appendix 1 [file jmir_v25i1e44795_app1.docx]

**Supplementary Materials**

**Catalogue**

**Figure legends**  1

**Part I:** SARS-CoV-2 RNA detection in the surgical mask 2

**Part II:** The design concept of masklect 4

**Part III:** The design concept of mask management machine 6

**Part IV:** The design concept of voiceprint health code 9

**Part V:** Mathematical simulations after MV-STRONG system running 11

**Reference** 19

**Figure legends**

**Figure S1.** Front view of the ear-loop masklect. **1-**Mask body, **2-**The nasal bridge above the mask, **3-**The ear strap.

**Figure S2.** A sectional view of masklect (front view). **4-**The skin-friendly fabric inner layer. **5, 6**-Composite multi-function filter layer. **7-**The external layer.

**Figure S3.** Schematic diagram of masklect filter (side view). **4-**The skin-friendly fabric inner layer. **5, 6**-Composite multi-function filter layer. **7**-The external layer. **a-**Sphacelus. **b-**Bacteria. **c-**Water vapor. **d-**SARS-CoV-2.

**Figure S4.** A holistic view of mask management machine (MMM). **1-**Component for mask collection. **2-**Component for mask detection. **3-**Operation panel. **4-**Slot for a new mask. **5-**Slot for the used mask. **6-**Window for QR code scanning. **7-**Window for barcode scanning.

**Figure S5.** Profile of the component for mask collection. **1-**Samples of the separated mask. **2-**Antigen detection. **3-**Nucleic acid detection. **4-**Results of nucleic acid detection. **5-**Results of antigen detection. **6-**Probes for signal reception.

**Figure S6.** Mask management machine network connections.

**Figure S7.** The construction of a voiceprint database and the process of voiceprint recognition. Abbreviations: MFCC, Mel-Frequency Cepstral Coefficients.

**Figure S8.** In the SEIN_h_AR model, a graphical scheme represents the relationships among different phases of infection.

**Figure S9.** **A.** Simulation of SEIN_h_AR model without operating the MV-STRONG system. **B.** Simulation of the SEIN_h_AR model under the conditions of operating the MV-STRONG system. **Note:** The coordinates ***(x, y)*** of the blue dots in the figure represent ***(day, count)***, which refers to the time and count when the peak was reached.

**Figure S10.** Comparison of simulations at different application ratios of the MV-STRONG system.

**Part I SARS-CoV-2 RNA detection in the surgical mask**

Reverse transcription polymerase chain reaction-based SARS-CoV-2 RNA detection from respiratory samples (eg, nasopharynx) is the standard for diagnosis, the digestive tract has also been reported. ^4^ The mask collects and filters samples from the upper or lower respiratory and digestive tract, thereby retaining virus particles for detection. ^11^ Has now been confirmed in the ordinary surgical masks,^12,13^ N95,^14,15^ and improved masks detected virus RNA,^16,17^ and we performed trial included nine COVID-19 cases from China by random, the patient wears masks for different times with speeches, activities, singing, etc., nasopharyngeal swabs were taken in the same period as a comparison, the results in ***Table S1***.

The studies involving human participants were reviewed and approved by the Hospital Institutional Review Board of the Second Xiangya Hospital. Written informed consent was obtained from all subjects.

**Table S1:** Clinical and demographic characteristics of nine patients with COVID-19 and their Ct values of nasopharyngeal swabs and surgical masks.

| **Case** | **Sex/age(year)** | **Hospital stays** | **Symptoms** | **Nasopharyngeal**  **swab Ct values** | **Surgical mask Ct values** | **Time of mask use** | **Outcome** |
| --- | --- | --- | --- | --- | --- | --- | --- |
| 1 | 45/F | 30 | Coughing | N gene: 36.81  E gene: 35.50  Orf-1AB: 40.34 | N gene: 36.04  E gene: 24.24  Orf-1AB: 38.89 | 4 h | Survived |
| 2 | 30/F | 21 | Fever,  Coughing | N gene: 35.54  E gene: 26.80  Orf-1AB: 37.71 | N gene: 24.40  E gene: 24.58  Orf-1AB: 28.01 | 5 h | Survived |
| 3 | 19/M | 19 | Fever,  Anosmia | N gene: 34.24  E gene: 30.82  Orf-1AB: 34.33 | N gene: 37.07  E gene: 26.24  Orf-1AB: 41.25 | 6 h | Survived |
| 4 | 12/M | 16 | Anosmia,  Dry coughing | N gene: 39.49  E gene: 28.38  Orf-1AB: 39.38 | N gene: 23.59  E gene: 25.23  Orf-1AB: 25.95 | 12 h | Survived |
| 5 | 50/M | 30 | Fever,  Fatigue | N gene: 37.39  E gene: 27.42  Orf-1AB: 36.72 | N gene: 37.43  E gene: 25.84  Orf-1AB: 40.09 | 11 h | Survived |
| 6 | 17/F | 25 | Fever, Dry coughing | N gene: 33.04  E gene: 25.95  Orf-1AB: 32.44 | N gene: 34.48  E gene: 25.11  Orf-1AB: 37.71 | 4 h | Survived |
| 7 | 63/F | 17 | Coughing,  Fatigue | N gene: 39.99  E gene: 39.08  Orf-1AB: 37.77 | N gene: 23.25  E gene: 24.78  Orf-1AB:25.92 | 8 h | Survived |
| 8 | 4/F | 21 | Asymptomatic infection | N gene: 38.77  E gene: 30.14  Orf-1AB: 39.10 | N gene: 38.53  E gene: 27.17  Orf-1AB:No | 3 h | Survived |
| 9 | 40/M | 20 | Fever, Dry coughing | N gene: 33.05  E gene: 25.38  Orf-1AB: 33.86 | N gene: 28.71  E gene: 23.26  Orf-1AB:31.6 | 3 h | Survived |

**Part II The design concept of mask-lect**

A novel functional mask that combines protection and collection capabilities is called masklect, which is shaped like a surgical mask or N95 (depending on the application) but should be distinguished from the common masks in widespread use today to avoid confusion. Masklect can be manufactured from synthetic or natural polymers or composites, usually polypropylene (PP), polyethylene (PE), cellophane, felt or other biological materials.^5^ The overall structure is like that of a conventional mask, consisting of a mask body, a nasal band, and ear hooks ***(Figure S1)***.

The mask body consists of an inner layer, an outer layer, and a middle layer for the main functions ***(Figure S2)***.

The function of the inner layer is skin-friendly, water absorption, and primary filtration, such as epithelial necrosis, food residues, and sticky secretions. The outer layer is hydrophobic, and its main functions are anti-fouling, shaping, etc. The middle layer, also known as the filter layer or functional layer, can be made up of multiple layers of single-function structures stacked on top of each other ***(Figure S3)***.

On the one hand, bacteria or viruses can attach to the middle layer and be filtered out by gravitational settling, inertial impact of fibers, fiber interception, diffusion, and electrostatic attraction, thus blocking the transmission chain of SARS-CoV-2.^5,18^ On the other hand, the special material of the filter layer should avoid the destruction of nucleic acid or protein of SARS-CoV-2 by physicochemical factors as much as possible, thus facilitating the automatic detection of the mask management machine. In addition, biodegradability and recyclability deserve to be seriously considered to contribute to the mitigation of environmental problems. To facilitate the use of special groups, more interesting and user-friendly features will be necessary to be considered, such as a moisture-sensitive color change to indicate the use level of the mask, and moisture-sensitive hardening or deformation to remind the blind to change the mask in time.


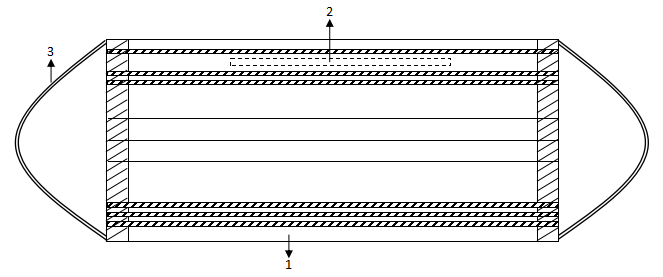


**Figure S1.** Front view of the ear-loop masklect. **1-**Mask body, **2-**The nasal bridge above the mask, **3-**The ear strap.


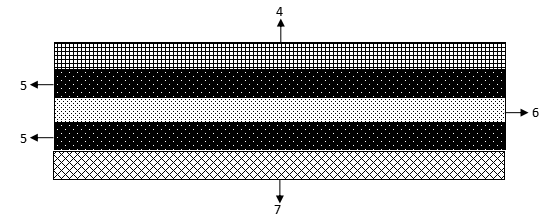


**Figure S2.** A sectional view of masklect (front view). **4-**The skin-friendly fabric inner layer. **5, 6**-Composite multi-function filter layer. **7-**The external layer.


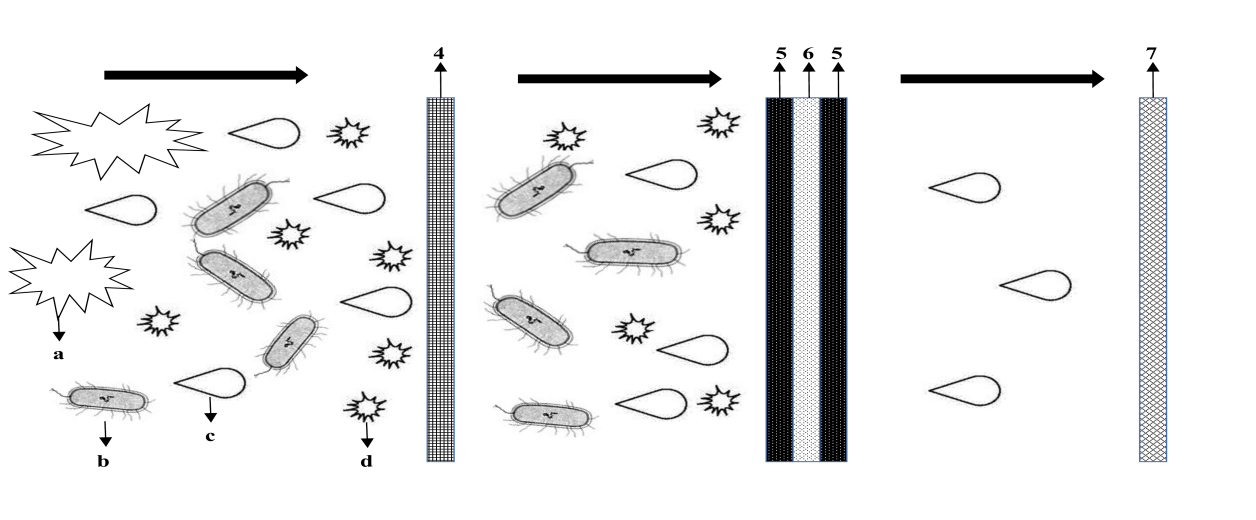


**Figure S3.** Schematic diagram of masklect filter (side view). **4-**The skin-friendly fabric inner layer. **5, 6**-Composite multi-function filter layer. **7**-The external layer. **a-**Sphacelus. **b-**Bacteria. **c-**Water vapor. d-SARS-CoV-2.

**Part III The design concept of the mask management machine**

A matching mask management machine (MMM), like an ATM, is scattered throughout streets and communities to collect, detect, and replace the used masks. The MMM consists of a collection component, a detection component, an operation panel, a mask slot, and windows for scanning ***(Figure S4)***.

Discarded masks are pre-processed in the collection component, where they are primarily disassembled into elastic and metal strips and mask bodies. The mask body that is sent to the detection component will be divided into two parts from the middle. One part of the sample will be used for the nucleic acid detection of SARS-CoV-2, and the other for antigen detection. The test results of all samples will be acquired by the signal reception probe and transmitted to the smart terminal ***(Figure S5)***. All disassembled elastic and metal strips and mask bodies will be separated, packaged and delivered to a designated location for centralized disinfection and sterilization before further reprocessing.

The MMM intelligent terminal accesses information about the user's mask detection and uploads the latest results to the cloud. Users and intelligent terminals can download test results for updating information at any time. The cloud can integrate other data sources to improve compatibility. Other users can upload and update information about COVID-19 via the local area network or download the protective and travel advice directly from the cloud ***(Figure S6)***.

**
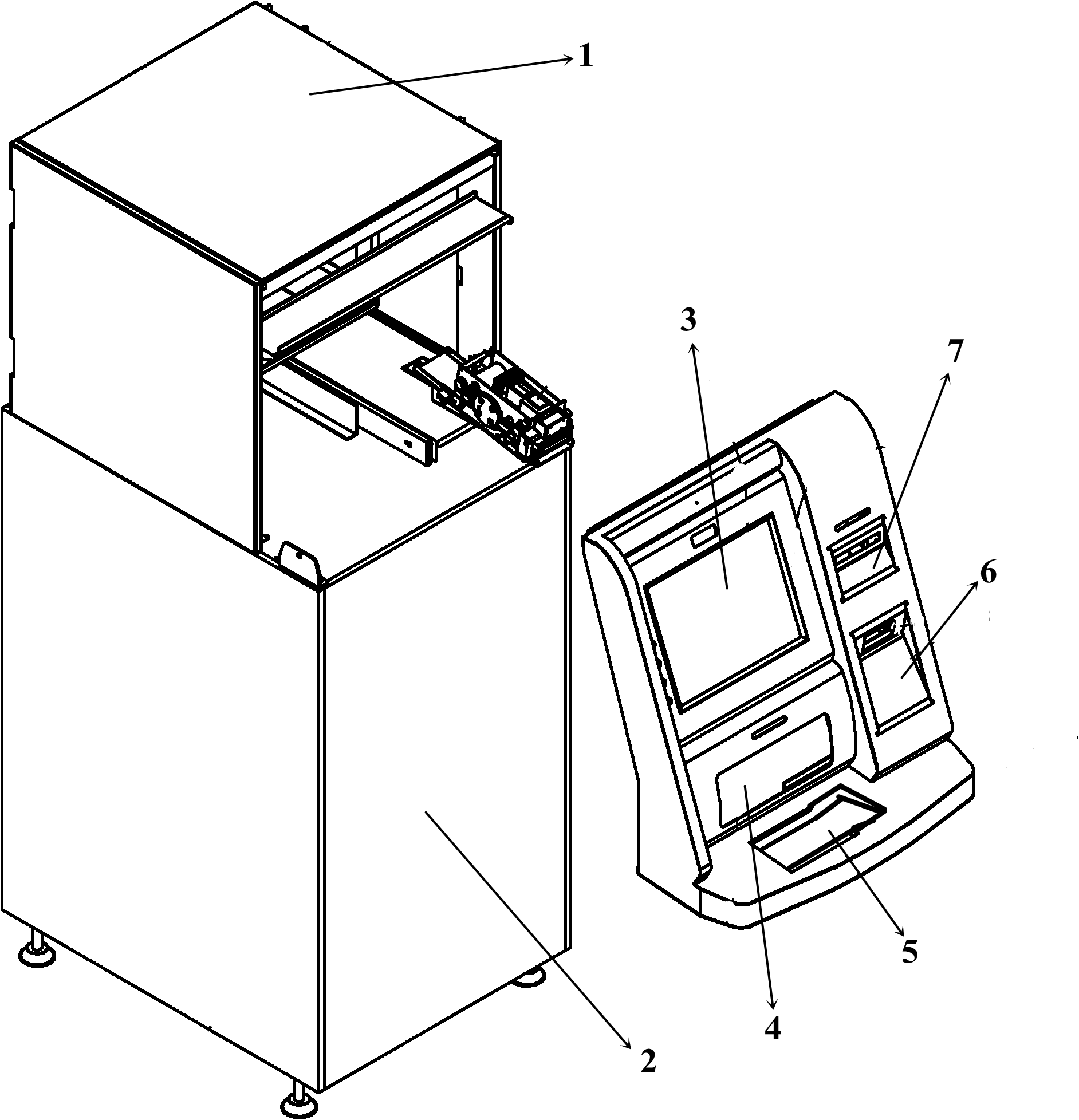
**

**Figure S4.** A holistic view of mask management machine (MMM). **1-**Component for mask collection. **2-**Component for mask detection. **3-**Operation panel. **4-**Slot for a new mask. **5-**Slot for the used mask. **6-**Window for QR code scanning. **7-**Window for barcode scanning.


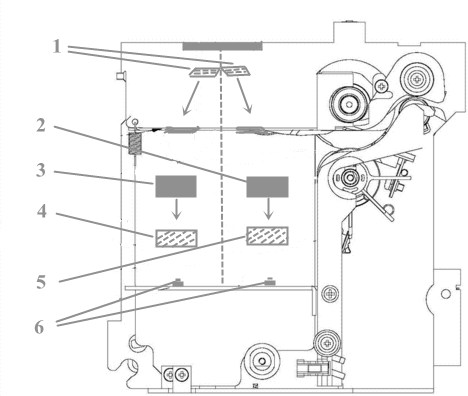


**Figure S5.** Profile of the component for mask collection. **1-**Samples of the separated mask. **2-**Antigen detection. **3-**Nucleic acid detection. **4-**Results of nucleic acid detection. **5-**Results of antigen detection. **6-**Probes for signal reception.


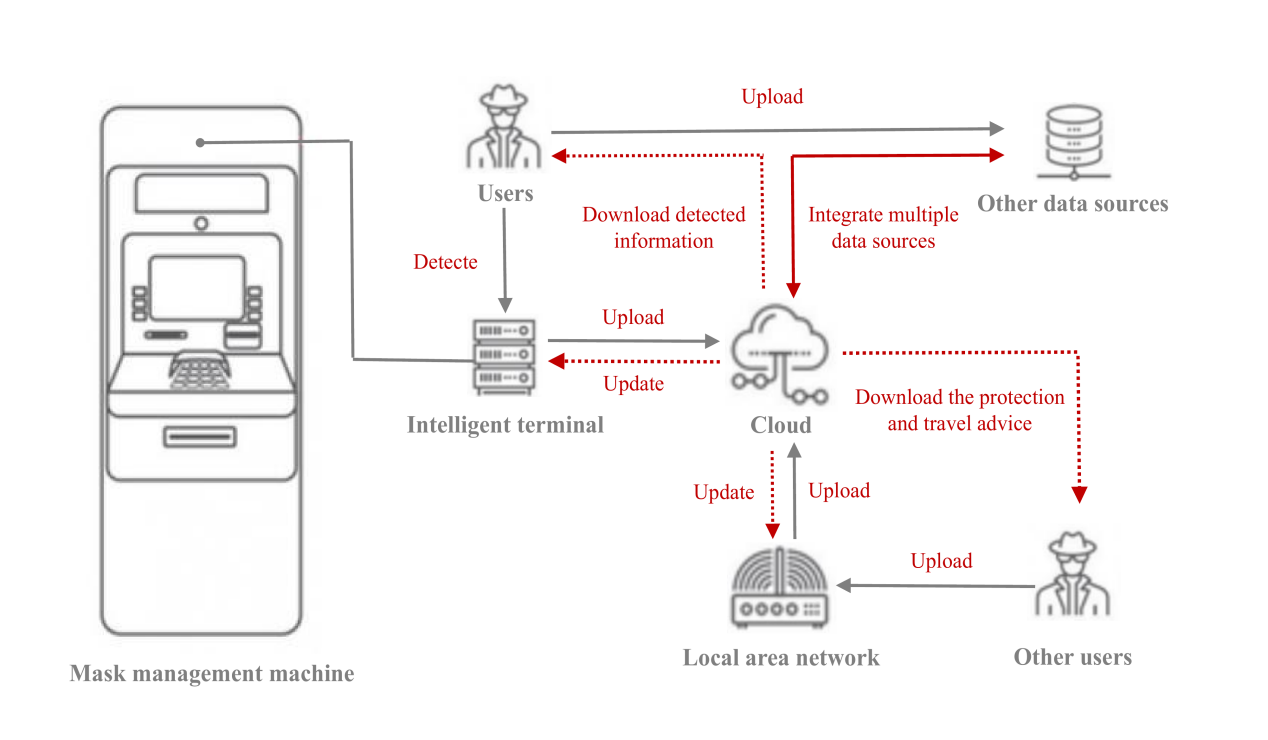


**Figure S6.** Mask management machine network connections.

**Part IV The design concept of voiceprint health code**

To detect those infected with COVID-19 as early as possible, especially those are slight symptoms, it makes sense to monitor the changes in voiceprints before and after infection by an intelligent voiceprint health code (***Figure S7***). Users record their audio, mainly coughs, via a smartphone app and convert it into an input spectrum. It is recommended to use repeated moderate coughs three times for the detection of voiceprint changes before and after recording and validation to facilitate voiceprint homogenization.The system then accomplishes speech feature extraction and forms a deep speech system using the Mel-Frequency Cepstral Coefficients (MFCC). After that, it is converted into speaker vectors and integrated with the user's GPS and GEO.AI information, which is collectively constructed into the voiceprint database.

Users can upload their audio to the voiceprint database for matching and testing at any time. These tests are mainly about the changes in their voiceprints and the company of time and space with those whose voiceprints have changed. Through the system's intelligent computing and integrated analysis, it is easy to discriminate groups with changed voiceprint and track their activity trajectory more quickly, which will greatly shorten the time gap for tracing the source of infection. As a complement to mask detection, this self-testing program will promote epidemic prevention towards intelligence, non-invasiveness, convenience, and low cost, and will better achieve mass prevention and economic recovery.

**
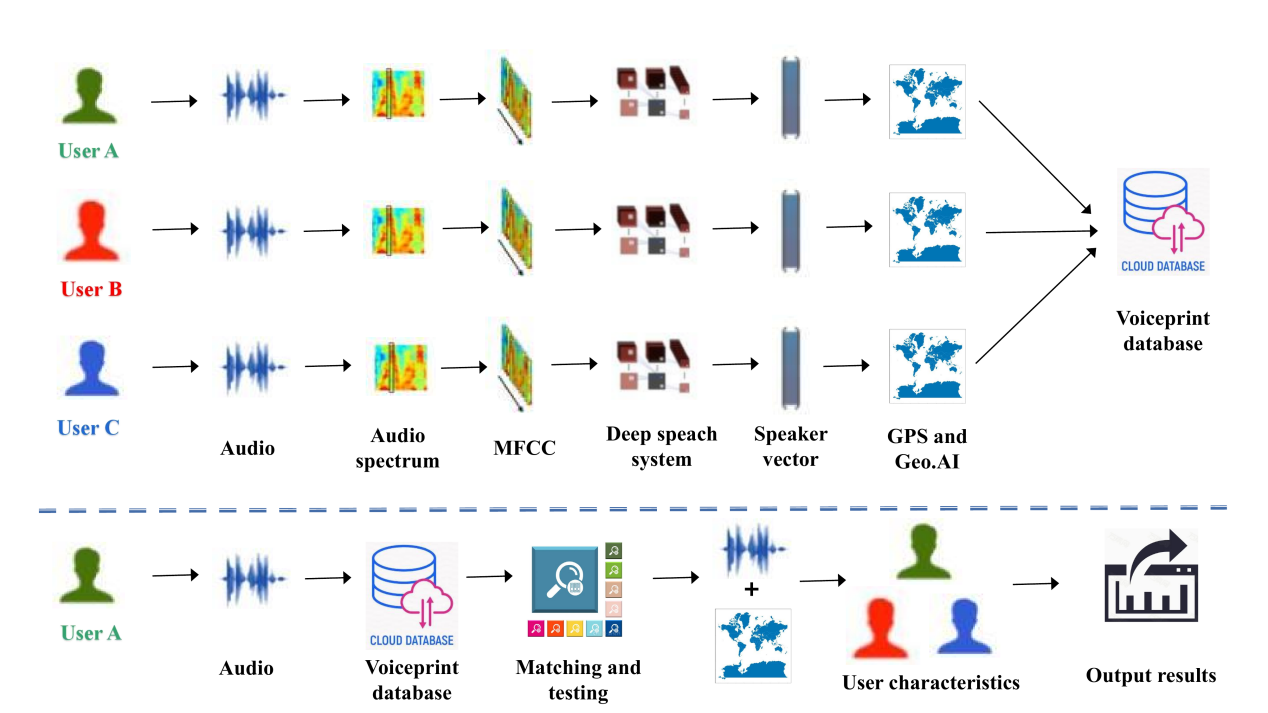
**

**Figure S7.** The construction of a voiceprint database and the process of voiceprint recognition. Abbreviations: MFCC, Mel-Frequency Cepstral Coefficients.

**Part V Mathematical Simulations for MV-STRONG system running**

**PURPOSE:** To better theoretically verify the feasibility of the novel system, we evaluated the trends in the number of various groups by using mathematical simulations and epidemiological models under both operational and non-operational conditions of the MV-STRONG system.

**METHODS:** we extend and complement the classic SE(A)IR model (called SEIN_h_AR), and proposed a novel mean-field epidemiological model for COVID-19 using the least-squares method to estimate the parameters.^19,20^ We consider a large-scale metapopulation model describing a set of cities with a baseline two million population connected by human mobility, the whole population is subdivided into the COVID-19-relevant six epidemiological compartments, which are: susceptible (S, uninfected), exposed (E, contaminated but not infected due to the incubation period), undetected infection (I, asymptomatic or mildly symptomatic), confirmed infection (N_h_, notified and hospitalized infection), confirmed asymptomatic (A, identified asymptomatic infection), and recovering individuals (R, recovered) ( the SEIN_h_AR model, see ***Figure S8*** for details).


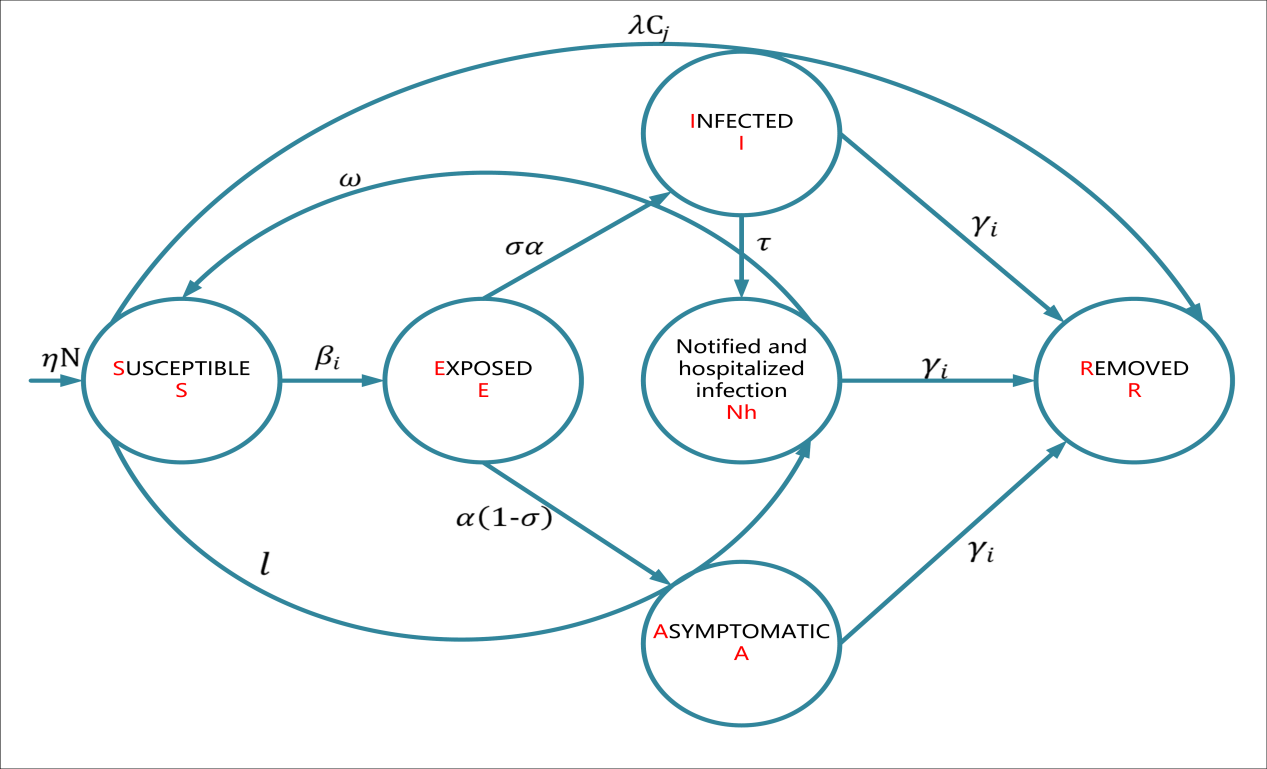


**Figure S8.** In the SEIN_h_AR model, a graphical scheme represents the relationships among different phases of infection.

**PROCESSES:**

# Mathematical Simulation before MV-STRONG System Running

$$\begin{aligned} \frac{dS_{before}\left( t \right)}{dt}= \eta N- \frac{\beta_{i}S_{before}\left( t \right)\left( E_{before}\left( t \right)+A_{before}\left( t \right) \right)}{N-N_{hbefore}}-\sum_{j=1}^{4} C_{j}\lambda S_{before}\left( t \right)\# \left( 1 \right) \end{aligned}$$

$$\begin{aligned} \frac{dE_{before}\left( t \right)}{dt}= \frac{\beta_{i}S_{before}\left( t \right)\left( E_{before}\left( t \right)+A_{before}\left( t \right) \right)}{N-N_{hbefore}}-\left( \sigma+\eta\right)*E_{before}\left( t \right)\# \left( 2 \right) \end{aligned}$$

$$\begin{aligned} \frac{dI_{before}\left( t \right)}{dt}= \sigma\alpha E_{before}\left( t \right)-\left( \gamma_{i}+\eta\right)*I_{before}\left( t \right)\# \left( 3 \right) \end{aligned}$$

$$\begin{aligned} \frac{dN_{h}\left( t \right)}{dt}= \tau I_{before}\left( t \right)-\left( \gamma_{i}+\eta\right)*N_{h}\left( t \right)\# \left( 4 \right) \end{aligned}$$

$$\begin{aligned} \frac{dA_{before}\left( t \right)}{dt}= \sigma\left( 1-\alpha\right)E_{before}\left( t \right)-\left( \gamma_{i}+\eta\right)*A_{before}\left( t \right)\# \left( 5 \right) \end{aligned}$$

$$\begin{aligned} \frac{dR_{before}\left( t \right)}{dt}= \gamma_{i}\left( I_{before}\left( t \right)+A_{before}\left( t \right)+N_{hbefore}\left( t \right) \right)+\eta*R_{before}\left( t \right)\# \left( 6 \right) \end{aligned}$$

# Mathematical Simulation after MV-STRONG System Running

$$\begin{aligned} \frac{dS_{after}\left( t \right)}{dt}= \eta N- \frac{\beta_{i}S_{after}\left( t \right)\left( E_{after}\left( t \right)+A_{after}\left( t \right) \right)}{N-N_{hafter}}- \\ \sum_{j=1}^{4} C_{j}\lambda S_{after}\left( t \right)-\eta*S_{after}\left( t \right)-\iota*S_{after}\left( t \right)\# \left( 7 \right) \end{aligned}$$

$$\begin{aligned} \frac{dE_{after}\left( t \right)}{dt}= \frac{\beta_{i}S_{after}\left( t \right)\left( E_{after}\left( t \right)+A_{after}\left( t \right) \right)}{N-N_{hafter}}-\left( \sigma+\eta\right)*E_{after}\left( t \right)\# \left( 8 \right) \end{aligned}$$

$$\begin{aligned} \frac{dI_{after}\left( t \right)}{dt}= \sigma\alpha E_{after}\left( t \right)-\left( \gamma_{i}+\eta\right)*I_{after}\left( t \right)\# \left( 9 \right) \end{aligned}$$

$$\begin{aligned} \frac{N_{hafter}\left( t \right)}{dt}= \iota*S_{after}\left( t \right)+\tau*I_{after}\left( t \right)-\left( \omega+\eta\right)*N_{hafter}\left( t \right)-\left( \gamma_{i}+\eta\right)*N_{hafter}\left( t \right)\# \left( 10 \right) \end{aligned}$$

$$\begin{aligned} \frac{dA_{after}\left( t \right)}{dt}= \sigma\left( 1-\alpha\right)E_{after}\left( t \right)-\left( \gamma_{i}+\eta\right)*A_{after}\left( t \right)\# \left( 11 \right) \end{aligned}$$

$$\begin{aligned} \frac{dR_{after}\left( t \right)}{dt}= \gamma_{i}\left( I_{after}\left( t \right)+A_{after}\left( t \right)+N_{hafter}\left( t \right) \right)+\eta*R_{after}\left( t \right)\# \left( 12 \right) \end{aligned}$$

All of the parameters under consideration are positive integers denoted by Greek letters. The links between various stages of infection are shown in ***Figure S8***. The parameters are defined as follows:

The inflow rate (including birth and immigration) is represented by the parameter$\eta$. When a sensitive subject comes into contact with an exposed, infected, asymptomatic subject, the transmission rate $\beta_{i}$ (the probability of disease transmission in a single encounter multiplied by the average number of contacts per person) happens for each period$i$.

We suppose that vulnerable persons get the vaccination at a constant per capita rate$C_{j}\left( 0\leq C_{j}\leq1,j\in\left\{ 0,1,2,4 \right\} \right)$(i.e., vaccine coverage rate). The efficacy of immunization is$C_{j}\left( 0\leq\lambda\leq1 \right)$ . When$\lambda=0$, the vaccination has no effect, but it is effective when$\lambda=1$. The above parameters may be altered since they affect a variety of factors including government epidemic prevention measures, quarantine, and vaccines. The risk of infection from critically ill patients treated in proper ICUs is regarded to be minimal. $\alpha$and sigma represents the detection incubation rate in asymptomatic and symptomatic patients, respectively. These criteria represent the disease’s degree of attention and the number of tests done throughout the community; they may be improved by implementing a vast contact tracing. Furthermore, these characteristics determine the pace at which the exposed and asymptomatic populations get infected.

The average notification and hospitalization rate for symptomatic infection is $\tau$.

The $\iota$ is the average rate of those identified as vulnerable during the implementation of the MV-STRONG method at$\frac{1}{\omega}$ .

The rate of infected subjects being removed is denoted by $\gamma_{i}$; it may differ significantly if an appropriate treatment for the disease, such as the perfect test and vaccine efficacy at each specific period $i$, is known and adopted for diagnosed patients, but otherwise, they are likely comparable. This score may rise due to improved medicines and virus immunity.

- **Model fit for the SARS-CoV-2 epidemic before and after the MV-STRONG System**

We get the model parameters from the population size. The predicted parameter values are based on the number of presently infected persons with varied SOI (asymptomatic or paucisymptomatic, quarantined at home, approximately equivalent to variables $I\left( t \right)$ and $A\left( t \right)$in our model; before and after infection notification and hospitalization, approximately equivalent to variables$N_{hbefore}\left( t \right)$, and $N_{hafter}\left( t \right)$in our model, respectively. And the number of diagnosed persons who recovered with non-imperfect test accuracy (approximately equal to the amount$\int_{0}^{t} \gamma_{i}\left[ I\left( \psi\right)+A\left( \psi\right)+N_{hbefore}\left( \psi\right)+N_{hafter}\left( \psi\right) \right]d\psi$, that may be derived using our model).

We apply a best-fit approach to find the settings that minimize the sum of the squares of the errors locally. The model has many state variables as well as a large number of unknown parameters whose numerical computation is complex; an infinite number of unique parameter sets may be constructed, all of which fit the data equally well. In contrast, our parameters are control tuning knobs whose settings should correctly replicate the data. The model parameters were fitted via repeated local minimization of the sum of the squares of the errors, starting from a random initial estimate and based on a priori epidemiological and clinical information about relative parameter magnitude (as described above). During the simulation, the parameters were updated depending on the consecutive moves made by policymakers, each of increasing strength.

**RESULTS:**

1. **Comparison of non-operating and operating conditions**

Compared to the condition without running the MV-STRONG system ***(Figure S9)***, the simulation indicates that the novel system (1) allows the S to decline and the R to ascend at a faster rate and maintains the stability of S and R in the late stages of the outbreak to avoid resurgence. (2) advances the time to peak for the E, I, N_h_, and A to terminate the outbreak early, and reduces peak counts in each group to relieve medical stress. (3) also decreases the cumulative counts (comparison of area under the curve) of E, I, N_h_, and A to preserve vital health and minimize the impact of COVID-19.

1. **Comparison of different application ratios**

To some extent, efforts to promote this novel containment system are very challenging, and acceptance is often difficult to achieve overnight. Therefore, we attempted to compare the effects of the SEIN_h_AR model under different application ratios ***(Figure S10)***.

All things being equal, the MV-STRONG system advances the peak times of E, I, N_h_, and A, and ensures that fewer people are exposed and infected, thus flattening the transmission curve and shortening the duration of the outbreak. As the percentage of novel system applications gradually increases from 10% to 70% or even 100%, the speed of S descending and R ascending becomes faster, with peaks advancing earlier and outbreaks ending more quickly.


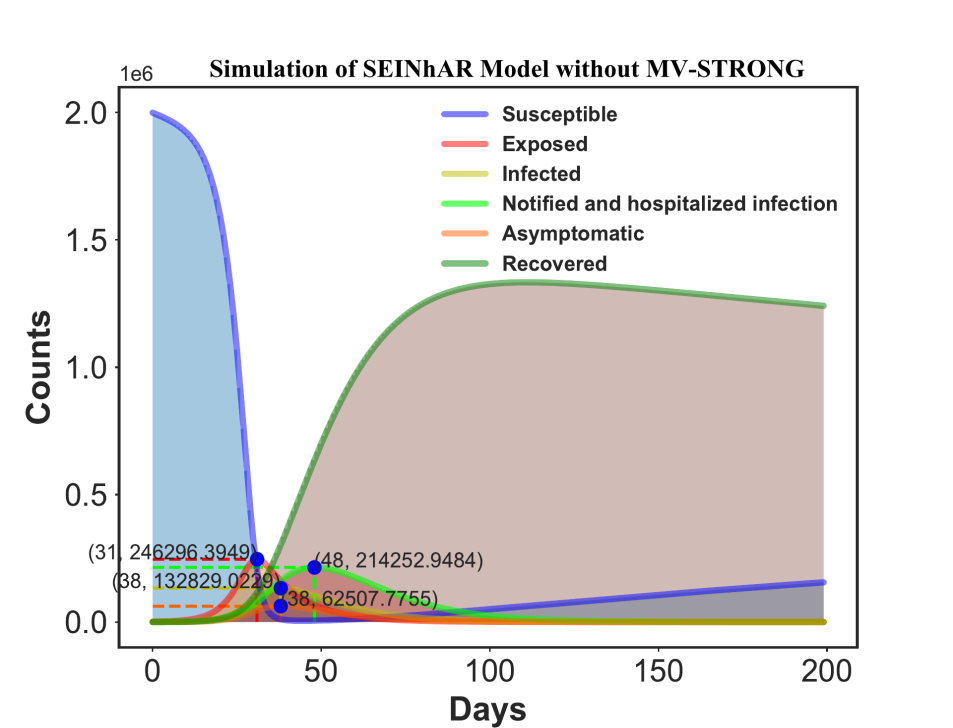

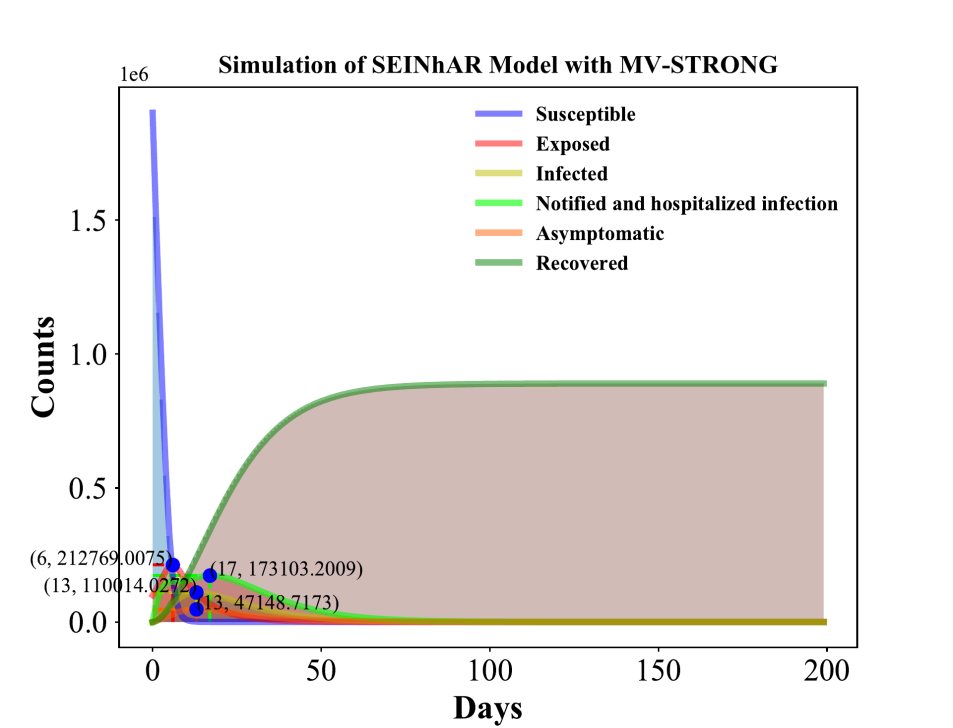


1. (B)

**Figure S9.** **A.** Simulation of SEIN_h_AR model without operating the MV-STRONG system. **B.** Simulation of the SEIN_h_AR model under the conditions of operating the MV-STRONG system. **Note:** The coordinates ***(x, y)*** of the blue dots in the figure represent ***(day, count)***, which refers to the time and count when the peak was reached.

| **Application** | **10 percentage** | **30 percentage** | **50 percentage** | **70 percentage** | **100 percentage** |
| --- | --- | --- | --- | --- | --- |
| **Non-**  **operating** | 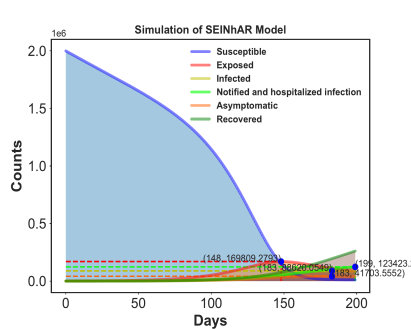 | 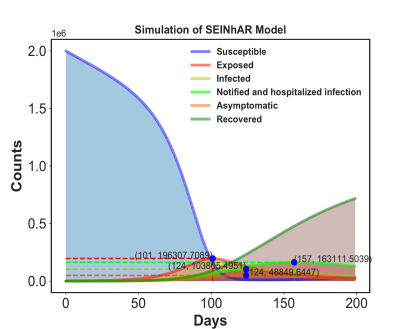 | 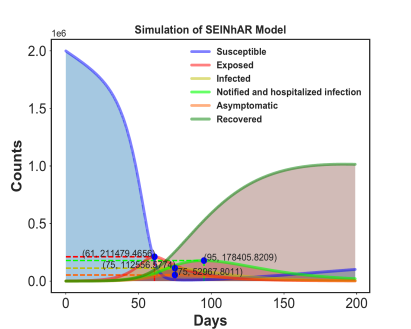 | 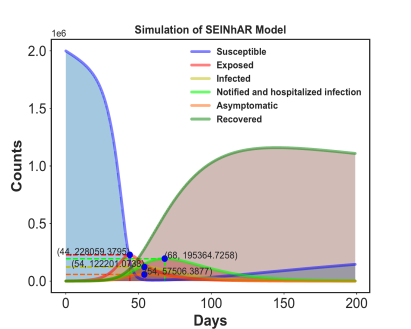 | 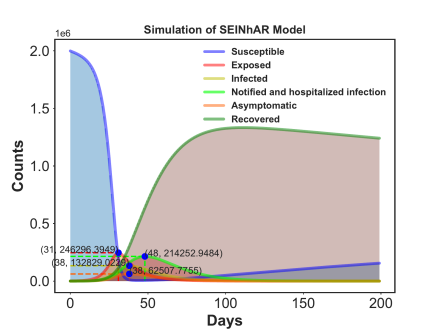 |
| **Operating** | 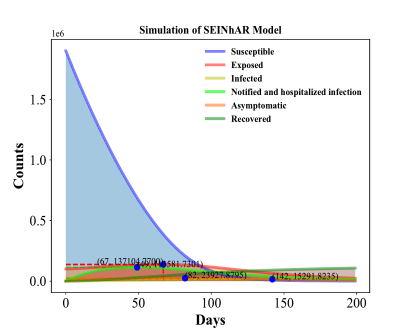 | 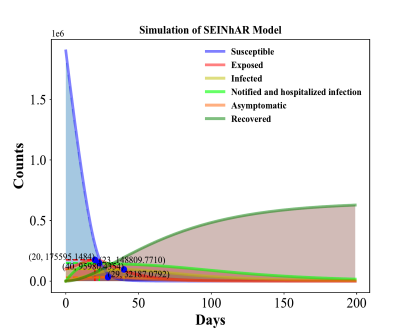 | 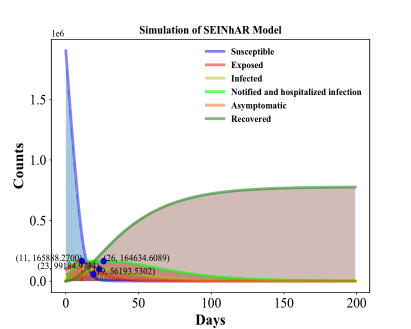 | 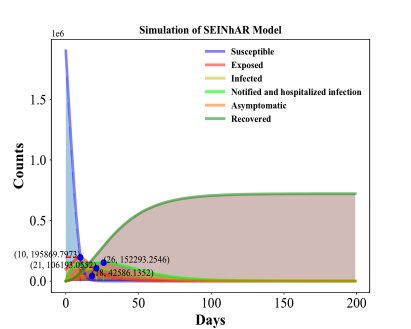 | 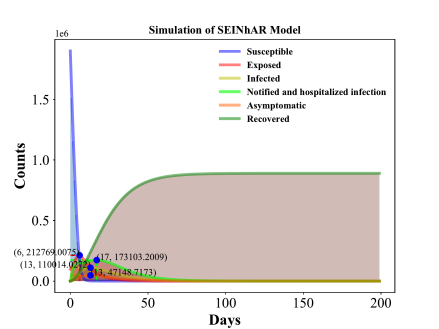 |

**Figure S10.** Comparison of simulations at different application ratios of the MV-STRONG system.

**DISCUSSION：**

As expected, the SEINhAR model accurately replicates and extends the classical SEI(A)R model of infectious diseases, while describing the spread regularity of COVID-19. From the simulation results, it seems that the MV-STRONG system will have great potential to become a globally centralized containment system. This novel system can terminate COVID-19 outbreaks early and reduce the number of infected patients, including pre-symptomatic, asymptomatic, slightly symptomatic, and unconfirmed. Moreover, this interesting effect will become more evident as the number of users grows and the adoption ratio gradually increase.

Regardless of outbreak or disease control, it seems meaningful to use this novel formation of epidemic prevention to reap the benefits. For one thing, the system's masklect, as a daily mask, can effectively block the spread of SARS-Cov-2 droplets and filter the aerosols produced by the virus, thus interrupting the transmission chain, which is in line with the current mainstream protection measure.^10,13^ For another, STRONG, as the framework of this system, is a fast-response intelligent digital contact tracking technology that can greatly minimize recall bias and infection identification interval.^8^ Not only that, the voiceprint health code, as a complementary analysis, can identify those infected with slight symptoms at an ultra-early stage. Compared to traditional testing and digital contact tracing, this combined system will be beneficial in restricting all types of transmitters, including asymptomatic, slightly symptomatic, pre-symptomatic, and multiple negative tests. The frequency of testing and the speed of reporting would be infinitely greater in this circular system.

From a global perspective of COVID-19 prevention and control, the MV-STRONG system is undoubtedly an effective measure to control infection. This proactive and continuous surveillance would be essential to guide the policy response and implementation of public health interventions.^9^ However, the premature arrival of peak infection will, in some sense, put pressure on the bed resources of social care. Consequently, as the promotion of this system is progressively increased, both large numbers of infected and suspected infections will be increasingly incremental. Healthcare investment and resource allocation should be prepared accordingly to cope with the peak wave of infections. While this investment, including the construction of the novel system, may be costly, the system, once in place, will fundamentally address the root causes of protracted outbreaks and resolve the current political and economic disruptions to restore normalcy to life.
